# Integrated Metaproteomics and Untargeted Metabolomics Reveal Season-Specific Enzyme Expression and Non-Volatile Metabolite Profiles in Medium-High-Temperature Daqu

**DOI:** 10.3390/foods15122181

**Published:** 2026-06-17

**Authors:** Qimai Wang, Xing Zheng, Xiaoli Gu, Qiuxiang Tang, Ping Song

**Affiliations:** 1 Jiangsu Co-Innovation Center of Efficient Processing and Utilization of Forest Resources, College of Chemical Engineering, Nanjing Forestry University, Nanjing 210037, China; wangqm111244@163.com (Q.W.); guxiaoli@njfu.edu.cn (X.G.); 2 State Key Laboratory of Microbial Technology, School of Food Science and Pharmaceutical Engineering, Nanjing Normal University, Nanjing 210023, China; jaa003722@163.com

**Keywords:** medium-high-temperature Daqu, seasonal variation, DIA-based quantitative metaproteomics, untargeted LC-MS metabolomics, carbohydrate-active enzymes, non-volatile flavour precursors, multi-omics integration

## Abstract

Seasonal fluctuations in open solid-state fermentation drive batch-to-batch variability in Chinese Baijiu Daqu; however, how environmental shifts reshape microbial functional expression and non-volatile flavour precursors in medium-high-temperature Daqu remains poorly resolved. In this study, data-independent acquisition (DIA)-based quantitative metaproteomics and untargeted liquid chromatography–mass spectrometry (LC-MS) metabolomics were integrated to characterise winter and summer Daqu from Luzhou, Sichuan. Among 2904 annotated non-volatile metabolites, orthogonal partial least squares discriminant analysis (OPLS-DA) revealed clear seasonal separation; 1472 differential metabolites (560 up- and 912 downregulated in winter vs. summer; variable importance in projection [VIP] > 1, *p* < 0.05) were enriched in glycolysis/gluconeogenesis, the tricarboxylic acid (TCA) cycle, amino acid biosynthesis, and starch/sucrose metabolism. DIA-based quantitative metaproteomics further resolved season-specific enzyme expression: summer Daqu exhibited elevated saccharolytic, glycolytic and amino-acid-converting enzymes (β-glucosidase, 6-phosphofructokinase, pyruvate dehydrogenase), whereas winter Daqu was enriched in glucose oxidase, phosphoenolpyruvate carboxykinase and aldehyde dehydrogenase, consistent with a pattern suggestive of carbon-storage prioritisation. Proteome–metabolome integration established a coherent “enzyme protein abundance–inferred metabolic tendency–metabolite accumulation” correlative framework axis: higher hydrolytic and central-carbon enzyme abundance in summer corresponded to increased maltose, lactate, acetate, L-glutamate and L-aspartate. Therefore, production season reshapes Daqu quality chiefly by corresponding to distinct patterns of in situ enzyme protein abundance, providing a DIA quantitative metaproteome-anchored mechanistic framework for screening high-expression starters and stabilising seasonal Daqu quality.

## 1. Introduction

In Chinese Baijiu production, Daqu functions as an indispensable saccharification and fermentation starter that harbours diverse microbial consortia and multi-enzyme systems collectively governing substrate degradation efficiency, precursor supply, and flavour development trajectories [1,2]. According to the peak internal temperature attained during brick fermentation, Daqu is classified into four types: high temperature (>60 °C), medium-high temperature (55–60 °C), medium temperature (50–55 °C), and low temperature (<50 °C) [3]. Even subtle variations in Daqu quality can cascade into substantial differences in the sensory profile of the final spirit, posing critical challenges for industrial-scale product consistency. Recent multi-omics investigations have progressively elucidated the “Daqu–microbiome–metabolism–flavour” continuum, demonstrating how microbial succession patterns correlate with organic acid dynamics and aroma biosynthesis phases during fermentation, thereby providing actionable process indicators for quality control [4]. Cross-regional studies further reveal significant compositional and functional heterogeneity among Daqu types, underscoring the necessity of environment-specific quality management strategies [5]. Nevertheless, persistent seasonal quality fluctuations in industrial Daqu production indicate that achieving the consistent “high-quality Daqu to high-quality Baijiu” transformation requires systematic, transferable evaluation frameworks specifically targeted to the Daqu manufacturing stage [6,7].

Daqu production employs open solid-state fermentation systems in which microbial community establishment depends critically on environmental parameters including workshop temperature, humidity, ventilation patterns, and brick-turning frequencies, all of which exhibit pronounced seasonal variations [8]. Summer conditions, characterised by elevated temperature and humidity, selectively enrich thermotolerant taxa such as *Bacillus* and *lactobacilli*, enhancing saccharification and organic acid production capacities. Conversely, winter environments generate stratified microenvironmental gradients—particularly in CO_2_ distribution—that affect community assembly patterns and precursor spatial heterogeneity, ultimately redirecting flavour development pathways [9]. Temperature perturbations fundamentally shape signature compound formation through adaptive microbial responses, with temperature–humidity fluctuation rates during heating phases exerting particularly strong directional selection pressure on community structure and metabolic function. This “environmental perturbation to community composition shift to altered metabolic output” cascade has been proposed as a primary ecological framework for understanding seasonal batch variations in Daqu, although the specific mechanistic links within this chain remain to be fully validated for medium-high-temperature systems [10,11]. Comprehensive industrial comparisons across strong-aroma, Fen-flavour, and sauce-aroma Daqu types confirm systematic seasonal divergence in community composition, enzymatic profiles, and flavour–metabolite correlations [12,13,14]. Consequently, seasonal environmental perturbations propagating through microbial metabolic networks and modulating functional outputs constitute the foremost challenge in Daqu quality standardisation [15].

Non-volatile metabolites—including amino acids, peptides, saccharides, organic acids, and lipids—represent fundamental precursors of the volatile compounds that define Baijiu character, such as esters, pyrazines, and Maillard reaction products [16]. Within Daqu matrices, non-volatile profiles document substrate degradation and metabolic processes and may therefore serve as sensitive early-stage quality indicators. Multi-omics studies of medium-temperature Daqu have demonstrated lactic acid dominance during early fermentation, coinciding with bacterial-to-fungal succession and suggesting that organic acid profiles can act as diagnostic windows into community transitions. High-temperature Daqu studies have proposed amino acid imbalance indices as quality metrics, with specific amino acids such as arginine exhibiting verifiable regulatory effects on downstream lactic acid and higher-alcohol formation, substantiating non-volatile-to-volatile causal relationships [17].

Non-targeted metabolomics platforms based on LC-MS, gas chromatography–mass spectrometry (GC-MS) and nuclear magnetic resonance (NMR) offer broad chemical coverage of fermentation networks and, when coupled with chemometric models such as principal component analysis (PCA), partial least squares discriminant analysis (PLS-DA) and OPLS-DA, have become a standard approach for biomarker discovery in Daqu and Baijiu research [18,19,20,21]. However, metabolomics alone captures the endpoint chemical phenotype rather than the functional drivers of metabolite accumulation, making it difficult to determine whether seasonal metabolite divergence arises from altered enzymatic capacity, shifted microbial composition, or substrate availability. Bridging this gap requires an additional functional layer that can directly report which catalytic activities are actually being expressed in situ.

Metaproteomics—particularly the recent generation of data-independent acquisition (DIA) workflows—has emerged as the most direct readout of in situ functional expression in complex fermentation microbiomes, since it quantifies the proteins that are actually synthesised rather than the genes that could potentially be expressed [12,13,22]. Compared with metagenomics or amplicon sequencing, metaproteomics circumvents the well-known mismatch between genetic potential and realised function; compared with classical enzyme assays, it provides simultaneous, system-wide quantification of hundreds of catalytic proteins, including the carbohydrate-active enzyme (CAZyme) repertoire that underpins solid-state saccharification. Coupling DIA metaproteomics with untargeted metabolomics therefore enables a causally interpretable “enzyme expression—substrate conversion—metabolite accumulation” axis to be reconstructed from a single set of fermentation samples, providing a framework for generating mechanistic hypotheses linking enzyme expression patterns to metabolite accumulation, which can guide future validation studies through direct enzyme activity assays and metabolic flux analyses [23]. To date, however, such an integrated proteome–metabolome analysis has not been reported for medium-high-temperature Daqu, whose temperature profile (peak 55–60 °C) and microbial composition differ markedly from those of Fen-flavour or high-temperature sauce-flavour Daqu, leaving the seasonal regulation of its functional enzyme landscape largely unexplored.

Therefore, the present study aims to: (i) profile the non-volatile metabolome of winter and summer medium-high-temperature Daqu by untargeted LC-MS metabolomics; (ii) resolve season-specific patterns of enzyme protein expression by DIA-based quantitative metaproteomics with Carbohydrate-Active enZYmes Database (CAZy)/Gene Ontology (GO)/Kyoto Encyclopedia of Genes and Genomes (KEGG) annotation; and (iii) integrate the two omics layers to identify candidate enzyme–metabolite associations that may underlie seasonal Daqu compositional divergence. The findings are intended to generate testable hypotheses and candidate functional enzyme markers for future validation through direct activity assays, pilot fermentation experiments, and volatile/sensory analysis of the final Baijiu product.

## 2. Materials and Methods

### 2.1. Materials

Medium-high-temperature Daqu samples were collected in winter (December) and summer (July) from a Baijiu manufacturer in Luzhou, Sichuan Province, China. Sampling was conducted in December (winter; average ambient workshop temperature of 8–12 °C, relative humidity of 50–60%) and July (summer; average ambient workshop temperature of 32–38 °C, relative humidity of 70–85%). Despite these environmental differences, both winter and summer batches achieved peak internal fermentation temperatures of 55–60 °C through microbial bio-heat generation, classifying them as medium-high-temperature Daqu according to industry standards. For each season, Daqu bricks were sampled from three independent fermentation rooms. Within each room, three bricks were randomly collected from each of the upper, middle, and lower layers (9 bricks per room). The bricks from each individual fermentation room were pooled separately by mass and homogenised, yielding three independent biological replicates per season (*n* = 3 per season, 6 samples total). The pooled sample (‘Pool’) mentioned in Section 2.3.1 refers exclusively to an equal protein mixture of all 6 individual samples used solely for DDA spectral library construction and was not used for quantitative DIA analysis. Each biological replicate thus represents a single fermentation room’s composite sample, capturing the spatial heterogeneity within that room while maintaining room-to-room variation as the basis for biological replication. All bricks collected from the same season were pooled by mass, homogenised using a high-speed grinder, sealed and stored at 4 °C until further analysis. LC-MS-grade reagents and trypsin were purchased from Macklin Biochemical (Shanghai, China). Coomassie Brilliant Blue R-250, SDS, and Tris-HCl were purchased from Aladdin Reagent (Shanghai, China).

### 2.2. Untargeted Non-Volatile Metabolomics

#### 2.2.1. Sample Preparation

Samples were retrieved from 4 °C storage, and 20 mg of each sample was accurately weighed into a 2 mL sterile centrifuge tube. Two steel beads and a 300 µL aliquot of pre-cooled methanol containing 5 ppm 2-chloro-L-phenylalanine (internal standard) were added; samples were vortexed for 30 s and ground in a high-throughput tissue grinder at 55 Hz for 60 s, with grinding repeated once. Samples were sonicated in an ice bath for 10 min and then incubated at −20 °C for 30 min. After centrifugation at 12,000 rpm for 10 min at 4 °C, the supernatant was carefully collected, filtered through a 0.22 µm organic-phase membrane and transferred to autosampler vials. Given the exploratory nature of this study and the limited sample size (3 biological replicates per group), no independent pooled quality control (QC) sample was prepared for the routine relative standard deviation (RSD)-based filtering workflow.

#### 2.2.2. Chromatographic Conditions

Chromatographic separation was performed on a Thermo Vanquish Flex UHPLC (Thermo Fisher Scientific, Germering, Bavaria, Germany) system equipped with an ACQUITY UPLC HSS T3 column (100 Å, 1.8 µm, 2.1 mm × 100 mm, Waters Corporation, Milford, MA, USA) maintained at 40 °C, with a flow rate of 0.4 mL/min. Mobile phase A was 0.1% formic acid in water and mobile phase B was 0.1% formic acid in acetonitrile; the same gradient was used for both positive and negative ion modes. The gradient elution programme was: 0.0–1.0 min, 5% B; 1.0–4.7 min, linear ramp from 5% to 95% B; 4.7–6.0 min, 95% B; 6.1 min, returned to 5% B; and 6.1–8.5 min, 5% B for column re-equilibration. The autosampler temperature was 8 °C and the injection volume was 2 µL. Samples were injected in a randomised order to minimise instrumental drift effects.

#### 2.2.3. Mass Spectrometry Conditions

Detection was performed on a Thermo Q Exactive HF-X high-resolution mass spectrometer (Thermo Fisher Scientific, Bremen, Bremen, Germany), with 2-chloro-L-phenylalanine used as the internal standard for quantitative calibration. Data-dependent acquisition (DDA) was performed in both positive and negative ion modes, controlled by Xcalibur 4.7 software. A heated electrospray ionisation (HESI) source was applied with the following parameters: sheath gas of 40 arb, auxiliary gas of 10 arb, capillary temperature of 320 °C, auxiliary gas temperature of 300 °C, and spray voltage of 3.5 kV.

MS^1^ acquisition parameters: resolution of 60,000, scan range of 70–1000 *m*/*z*, AGC target set to Standard, and maximum injection time (max IT) of 100 ms. The top 10 precursor ions were selected for MS^2^ fragmentation with a 4 s dynamic exclusion; MS^2^ parameters were set as: resolution of 15,000, HCD collision energy of 30%, AGC target set to Standard, and max IT set to Auto.

#### 2.2.4. Data Processing, Metabolite Identification

Raw MS data were first converted to mzXML format using ProteoWizard (v3.0). MS-DIAL (v4.9.221218) was used as the sole data processing platform for all subsequent analyses, including peak detection, chromatographic alignment, feature extraction, normalisation, gap-filling and feature filtering. Features undetectable in more than 50% of the biological replicates within each seasonal group were excluded from further analysis; missing values were imputed using the built-in gap-filling algorithm of MS-DIAL.

Metabolite identification was performed by matching experimental spectra against the in-house PSNGM authentic standard library, as well as public databases including mzCloud, LIPID MAPS, HMDB, MoNA and NIST_2020_MSMS. Matching thresholds were set as: MS^1^ mass tolerance < 0.01 Da, MS^2^ mass tolerance < 0.05 Da, mass slice width 0.05 Da, and MS/MS spectral matching score ≥ 70. All annotations were manually curated to eliminate isomer ambiguity, adduct misassignment and biologically implausible matches; only metabolites meeting Metabolomics Standards Initiative (MSI) Level 2 or higher annotation confidence were retained for downstream analysis.

Data reliability and system stability were verified through the following three complementary approaches. (i) High biological reproducibility: PCA analysis based on all 2904 annotated metabolites showed tight clustering of samples within each seasonal group, with Pearson correlation coefficients > 0.85 between intra-group samples, indicating that biological variation far exceeded technical noise. (ii) Consistency with established metabolic patterns: The identified 1472 differential metabolites and enriched pathways (e.g., glycolysis, TCA cycle, amino acid metabolism) formed a coherent biological narrative. The observed seasonal patterns (e.g., higher levels of maltose, lactate and amino acids in summer Daqu) were consistent with known temperature-dependent microbial metabolic strategies, supporting the biological validity of the dataset. (iii) Cross-omics consistency: Key metabolomic findings were independently supported by the DIA quantitative metaproteomic data. For example, the higher abundance of glycolytic and glycoside hydrolase enzymes in summer Daqu corresponded to the accumulation of their related metabolites, a correlation that is highly unlikely to occur with poor-quality metabolomic data. Mass accuracy < 10 ppm and MSI Level 2 annotation confidence further supported the reliability of metabolite identification.

### 2.3. DIA-Based Quantitative Metaproteomic Analysis of Medium-High-Temperature Daqu

#### 2.3.1. Protein Extraction and Tryptic Digestion

Winter and summer Daqu samples were retrieved from 4 °C storage. Each sample was extracted with SDS–DTT–Tris-HCl (SDT) lysis buffer (4% SDS, 100 mM Tris-HCl, pH 7.6), and protein concentration was determined using the bicinchoninic acid (BCA) assay. A 20 µg aliquot of each protein extract was mixed with 5× loading buffer, boiled for 5 min, and resolved on a 4–20% pre-cast SDS-PAGE gradient gel at 180 V for 45 min, followed by Coomassie Brilliant Blue R-250 staining. Equal protein amounts from all samples were pooled to construct a Pool sample for spectral library generation. All samples (including the Pool) were digested using filter-aided sample preparation (FASP) with trypsin. The digested Pool peptides were fractionated into 10 fractions using a Thermo high-pH reversed-phase fractionation kit (Thermo Fisher Scientific, Rockford, IL, USA). All fractions and individual sample peptides were desalted using C18 Stage Tips, lyophilised, and reconstituted in 40 µL of 0.1% formic acid; peptide concentrations were measured at OD_280_. iRT standard peptides were spiked into both the Pool and individual samples. Fractionated Pool peptides were used for data-dependent acquisition (DDA) library generation, and individual samples were analysed by data-independent acquisition (DIA).

#### 2.3.2. Mass Spectrometry Methods

Data-dependent acquisition (DDA) mode was used for spectral library construction, and the analysis was performed on an Easy-nLC 1200 nanoflow LC system coupled to a Q-Exactive HF-X mass spectrometer (Thermo Fisher Scientific, Bremen, Bremen, Germany). Peptides were loaded onto a C18 analytical column (Thermo Scientific, ES802; 1.9 µm, 75 µm × 20 cm) and eluted with a linear gradient of 0.1% formic acid in acetonitrile at 300 nL/min. MS^1^ scans (350–1800 *m*/*z*) were acquired at a resolution of 60,000 (@*m*/*z* 200) with an AGC target of 1 × 10^6^ and a maximum injection time of 50 ms; dynamic exclusion was set to 10 s. The top 20 precursor ions were selected for MS^2^ with an isolation window of 1.5 *m*/*z*, MS^2^ resolution of 30,000 (@*m*/*z* 200), AGC target of 1 × 10^5^, max IT of 50 ms, and HCD normalised collision energy of 30 eV.

Data-independent acquisition (DIA) mode was used for quantitative analysis of all individual samples, and the detection was performed on the same LC-MS platform in positive ion mode. MS^1^ scans (350–1800 *m*/*z*) were acquired at a resolution of 120,000 (@*m*/*z* 200) with an AGC target of 3 × 10^6^ and a maximum injection time of 30 ms. MS^2^ scans were acquired across 44 DIA windows at a resolution of 30,000 (@*m*/*z* 200), AGC target of 3 × 10^6^, max IT set at auto, and HCD collision energy of 30 eV, in profile mode.

#### 2.3.3. Data Analysis

DDA raw data were imported into Spectronaut (v14.4.200727.47784) to construct the spectral library, with peptide-to-protein matching against the corresponding species reference database. Search parameters: enzyme, trypsin; maximum missed cleavages, 1; fixed modification, carbamidomethyl (C); variable modifications, methionine oxidation and protein N-terminal acetylation. Identifications were filtered to a false discovery rate (FDR) of <1% at both peptide and protein levels. DIA data were analysed in Spectronaut with the same database and library, using dynamic iRT for retention time prediction, MS^2^ interference correction, and cross-batch normalisation, with results filtered at Q-value ≤ 0.01 (FDR < 1%). Differential protein screening employed the following criteria: fold change (FC) > 1.5 or <0.67 between winter and summer groups, combined with Q-value < 0.05 (Welch’s *t*-test with Benjamini-Hochberg correction). Missing values were handled using the Spectronaut default imputation strategy: proteins not detected in any replicate of a given group were assigned a value based on the lower 5% quantile of the overall intensity distribution to enable fold-change calculation.

#### 2.3.4. Bioinformatic Analysis

Enzyme protein sequences were aligned against the CAZy database (v10.0, E-value < 1 × 10^−5^) for carbohydrate-active enzyme annotation. Functional annotation and pathway enrichment were performed using GO and KEGG databases. Fisher’s exact test was used for enrichment analysis at both protein and taxonomic levels.

## 3. Results and Discussion

### 3.1. Overview of the Non-Volatile Metabolome of Medium-High-Temperature Daqu

**Figure 1:**
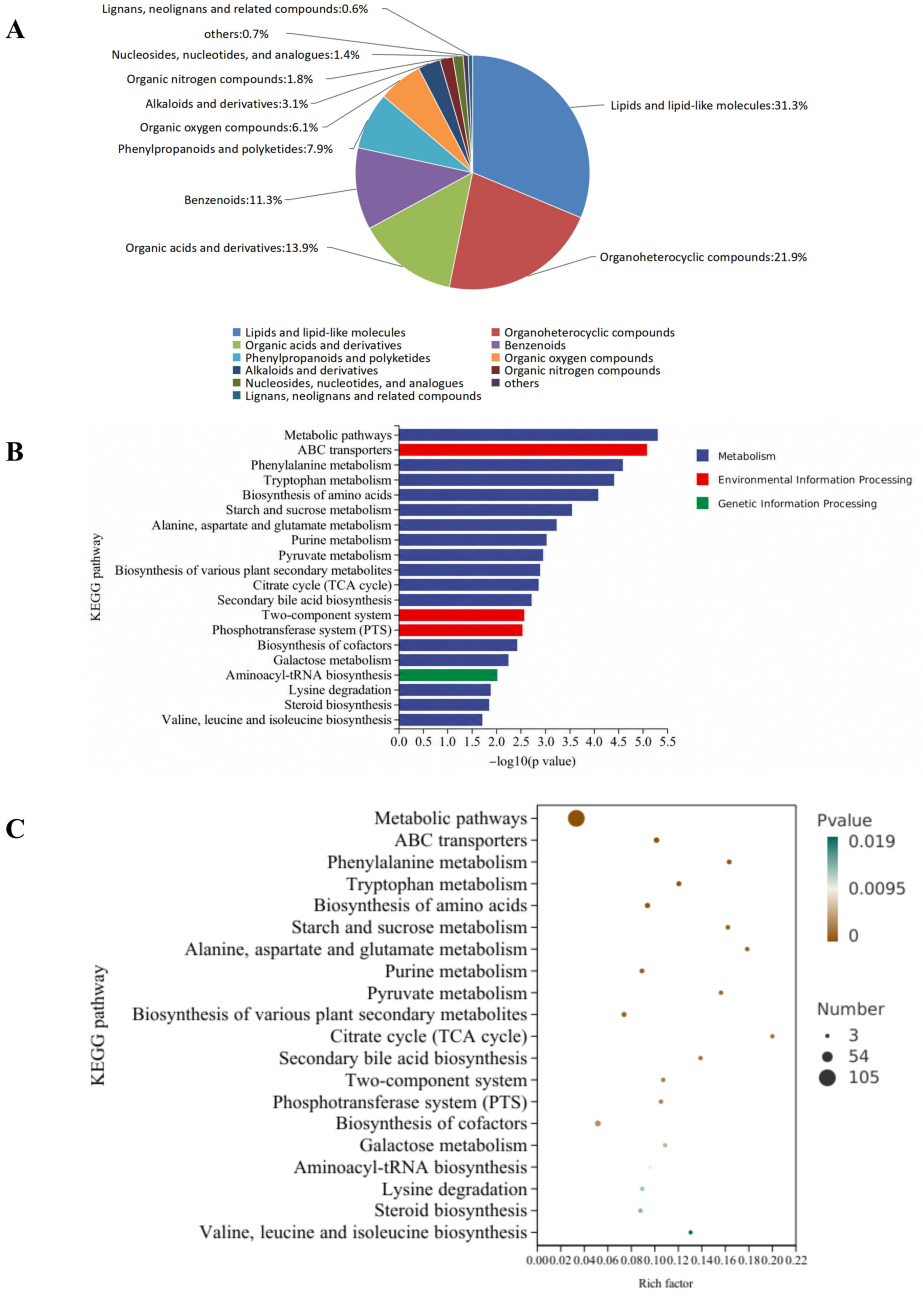
Classification of metabolites in two types of medium-high-temperature Daqu and summary of KEGG database annotation. (**A**) Classification of metabolites from two types of medium-high-temperature Daqu. (**B**) Bar chart of differential enrichment results. (**C**) Factor plot of differential enrichment results.

Untargeted metabolomic profiling of medium-high-temperature Daqu produced in two seasons identified 2904 non-volatile metabolites (Figure 1A): 1577 in positive ion mode and 1327 in negative ion mode. Lipids and lipid-like molecules (909), organic acids and derivatives (637), and organoheterocyclic compounds (405) constituted the three most abundant chemical classes, jointly accounting for 67.1% of the total.

The dominance of lipids and lipid-like molecules (31.3%) reflects both the lipid-rich composition of the wheat-based raw materials and the extensive lipolytic and oxidative biotransformation occurring during Daqu fermentation. Representative compounds include free fatty acids (e.g., linoleic acid, oleic acid, palmitic acid), lysophospholipids, phosphatidylethanolamines, sphingolipids, and fungal sterol derivatives (e.g., ergosterol). These compounds serve as direct precursors of ethyl esters (e.g., ethyl hexanoate, ethyl oleate) via esterification with ethanol during downstream Baijiu fermentation, and also modulate ester volatilisation rates through molecular interactions in the fermentation matrix.

Functionally, lipids and lipid-like molecules represent fundamental flavour-related substrates in medium-high-temperature Daqu, generating fatty acids and other aroma precursors via hydrolysis and oxidation. Sterols, vitamin E and related lipid derivatives also exert antioxidant activity that retards over-oxidation and excessive volatilisation of aroma compounds, thereby contributing to the flavour stability of the aged spirit. Organic acids and derivatives form another core component of the Daqu metabolic system: lactic acid, propionic acid and related compounds produced through microbial metabolism modulate the acidity of the fermentation environment, serve as essential flavour compounds and precursors of Baijiu aroma esters, and shape mouthfeel through downstream esterification reactions.

KEGG-based functional annotation (Figure 1B,C) showed that the identified non-volatile metabolites fell into three primary categories: metabolism, environmental information processing, and genetic information processing. At the secondary level, 20 pathways were represented, with carbohydrate metabolism and amino acid metabolism (each represented by 14 metabolites) showing the highest representation, followed by organic acid metabolism and biosynthesis of secondary metabolites (each represented by 12 metabolites). At the tertiary level, 116 pathways were enriched. Among them, ABC transporters showed the largest number of associated metabolites, followed by phenylalanine metabolism and tryptophan metabolism, indicating critical roles for transmembrane transport and aromatic amino acid metabolism in non-volatile flavour formation. ABC transporters mediate microbial uptake of nutrients and are central to maintaining metabolic activity in the fermentation matrix [24]. Phenylalanine metabolism directly links with the synthesis of phenylacetaldehyde and 2-phenylethanol, key contributors to the rose- and honey-like notes of Baijiu and major constituents of the so-called “pit-aroma complex” floral-fruity layer [25]. Tryptophan metabolism produces indole and other bioactive intermediates that act as precursors of higher alcohols and esters, enriching the complexity and layered character of Baijiu aroma [26].

Amino acid biosynthesis—the core pathway by which microorganisms produce the amino acids required for growth—provides not only nitrogen substrates for biomass accumulation but also precursors for downstream flavour compound formation [27,28]. Selected amino acids participate directly in Maillard reactions to generate pyrazines and related aroma molecules, or undergo decarboxylation and transamination to alcohols and aldehydes that constitute important elements of the Baijiu flavour pool [29,30]. Starch and sucrose metabolism, the central carbohydrate degradation route, progressively releases glucose and other monosaccharides, supplying carbon and energy for microbial metabolism while feeding into glycolysis and the TCA cycle to generate ethanol and organic acids that support the sweet, mellow body of Baijiu [31,32]. Purine metabolism contributes to nucleic acid biosynthesis and degradation, generates signalling molecules that regulate microbial activity, and yields nitrogen-containing heterocyclic compounds that imbue Baijiu with characteristic aged and roasted notes [33]. Pyruvate metabolism, downstream of glycolysis, produces ethanol, lactate and acetate that directly determine the alcohol-to-acid balance and overall harmony of the spirit [34].

The overall chemical-class distribution of the total metabolome is presented in Figure 1A; the season-resolved comparison highlighting significantly differential metabolites between winter and summer Daqu will be detailed in subsequent sections.

### 3.2. Seasonal Differences in the Non-Volatile Metabolome

#### 3.2.1. Principal Component Analysis

**Figure 2:**
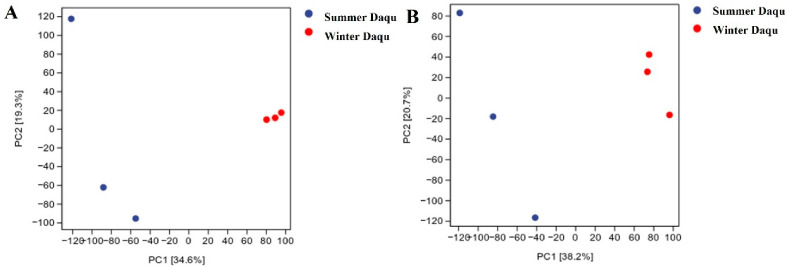
PCA plot of medium-high-temperature Daqu in different seasons. (**A**) Principal component analysis (PCA) plot in POS mode. (**B**) Principal component analysis (PCA) plot in NEG mode.

PCA of the 2904 annotated non-volatile metabolites was performed to assess overall sample variation and intra-group consistency (Figure 2A,B). Under positive ion mode, PC1 and PC2 jointly explained 55.9% of the variance (PC1 33.9%, PC2 22.0%); under negative ion mode, PC1 and PC2 explained 58.9% (PC1 36.9%, PC2 22.0%). Samples within each season clustered tightly while the two seasonal groups separated clearly, indicating good within-group consistency and pronounced seasonal divergence in non-volatile metabolite composition.

#### 3.2.2. OPLS-DA Modelling

**Figure 3:**
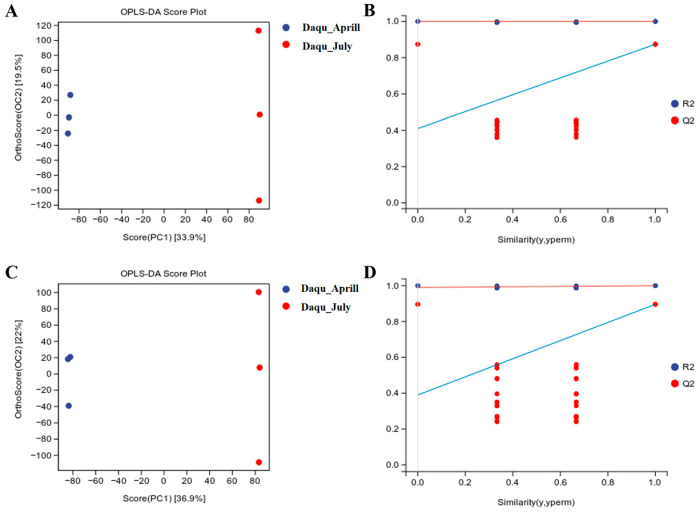
The blue regression line for R^2^Y, the red regression line for Q^2^Y. (**A**) OPLS-DA score plot in POS mode. (**B**) Model cross-validation plot in POS mode. (**C**) OPLS-DA score plot in NEG mode. (**D**) Model cross-validation plot in NEG mode.

OPLS-DA, a latent-variable regression method based on the covariance between predictor and response variables, identifies discriminating variables (VIP values) reflecting between-group differences. As shown in Figure 3A,C, samples from the two seasons were clearly separated within the 95% confidence ellipse, confirming distinct non-volatile metabolic profiles. As shown in Figure 3B,D, R^2^X, R^2^Y and Q^2^Y were used to evaluate model performance, reflecting the model’s capacity to explain the X and Y matrices and its predictive ability, respectively; values approaching 1 indicate stable and reliable models suitable for differential metabolite screening [35]. In the present study, the positive-ion-mode model yielded R^2^X = 0.534, R^2^Y = 1, and Q^2^Y = 0.874, while the negative-ion-mode model yielded R^2^X = 0.589, R^2^Y = 1, and Q^2^Y = 0.896, indicating that both OPLS-DA models were robust and suitable for downstream identification of seasonal differential metabolites.

#### 3.2.3. Differential Metabolite Screening

**Figure 4:**
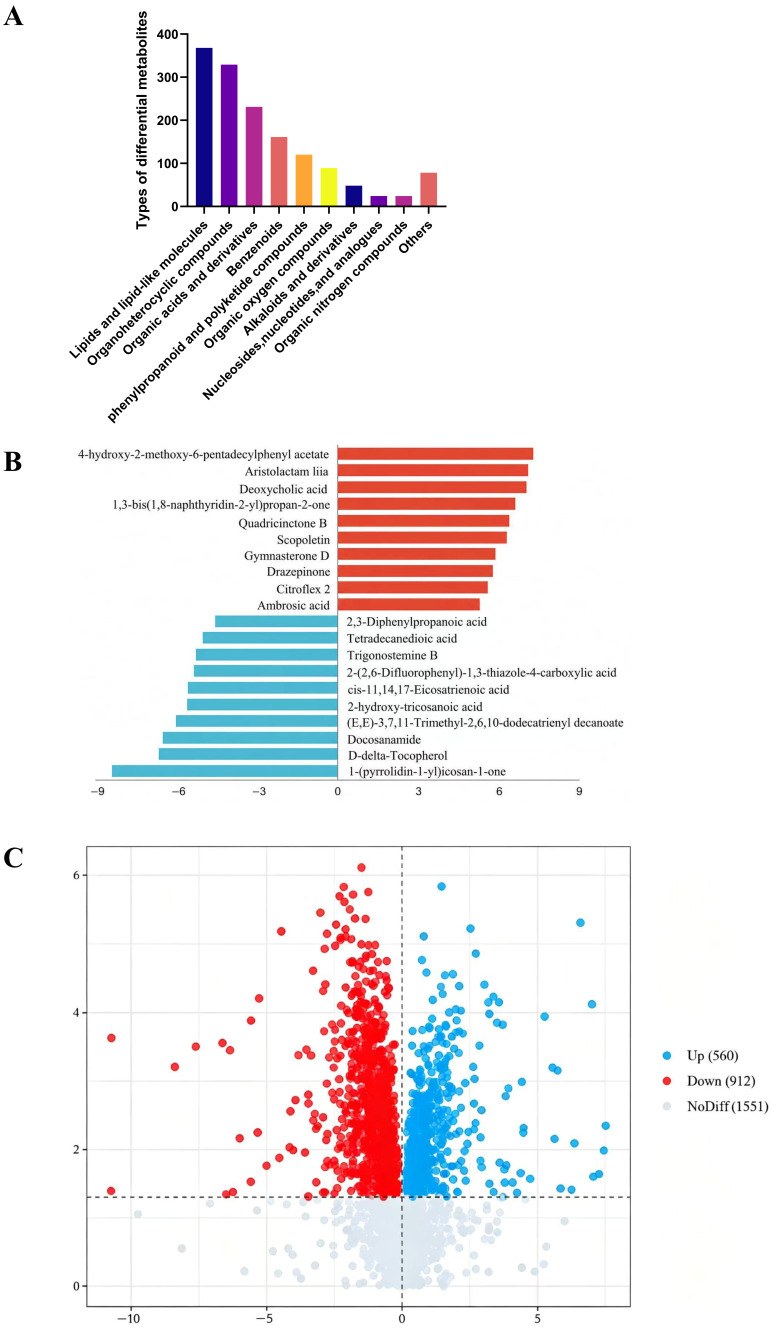
(**A**) Distribution of 1472 differential metabolites (VIP ≥ 1, *p* < 0.05) by chemical class in medium-high-temperature Daqu across different seasons. (**B**) Volcanic diagram of differential metabolites. (**C**) Upregulation map of differential metabolites in different seasons.

Based on the OPLS-DA outputs, candidate differential metabolites were initially selected by VIP value, then refined by univariate significance (*p* value) and fold change (FC). Using thresholds of VIP ≥ 1 and *p* < 0.05, a total of 1472 differential metabolites were identified (Figure 4A). As a subset of the 2904 total annotated metabolites shown in Figure 1A, these metabolites were predominantly distributed among lipids and lipid-like molecules, organoheterocyclic compounds, and organic acids and derivatives.

A volcano plot was used to visualise seasonal differences and to classify metabolites by direction of change (Figure 4B). Compared with summer Daqu, winter Daqu showed 560 upregulated and 912 downregulated metabolites, with downregulated species predominating overall. As shown in Figure 4C, upregulated metabolites were concentrated in lipids and lipid-like molecules and organoheterocyclic compounds, followed by organic acids and derivatives, benzenoids, and phenylpropanoids and polyketides; lower numbers were found in organic oxygen compounds, alkaloids and derivatives, nucleosides and analogues, and organic nitrogen compounds; lignans and related compounds (6) and single representatives of organometallic and hydrocarbon classes were also identified. Downregulated metabolites showed broader chemical-class distribution, with organoheterocyclic compounds and lipids and lipid-like molecules dominating, followed by organic acids and derivatives, benzenoids, and phenylpropanoids and polyketides; lower numbers occurred in organic oxygen compounds, alkaloids, organic nitrogen compounds, nucleosides, and lignans, with single hits for organosulphur and hydrocarbon classes. Overall, winter Daqu was characterised by a downregulation-dominant differential profile and broader chemical-class diversity among the downregulated species.

To highlight the most prominent contributors, the top 10 upregulated and top 10 downregulated metabolites were ranked by log-transformed FC. Compared with summer Daqu, the three most upregulated metabolites in winter Daqu were 4-hydroxy-2-methoxy-6-pentadecylphenyl acetate, aristolactam IIIa, and deoxycholic acid, while the three most downregulated were 1-(pyrrolidin-1-yl)icosan-1-one, D-δ-tocopherol, and docosanamide. Notably, several plant-derived or pharmacological metabolite annotations (e.g., aristolactam IIIa) likely reflect database-level isomer ambiguity in untargeted MS^2^ matching and should be regarded as putative annotations (MSI Level 2) requiring authentic-standard verification in future work.

The observed seasonal differences in non-volatile metabolite profiles likely arise from the interplay of multiple mechanisms rather than microbial biosynthetic pathway differences alone. First, differential microbial community composition between seasons—with thermotolerant bacteria (e.g., *Bacillus*, *Lactobacillus*) dominating in summer and psychrotolerant fungi (e.g., *Aspergillus*, *Rhizopus*) more prominent in winter—drives distinct enzymatic repertoires and thus different biosynthetic outputs. Second, non-enzymatic chemical reactions are temperature-dependent and contribute substantially to metabolite divergence: higher summer temperatures accelerate Maillard reactions between reducing sugars and amino acids, generating Amadori compounds and heterocyclic derivatives; promote lipid oxidation yielding aldehydes and secondary oxidation products; and enhance ester hydrolysis–esterification equilibria. Conversely, the lower temperatures of winter may favour the accumulation of intact lipids and reduce the rates of spontaneous oxidation and condensation reactions. Third, substrate accessibility differs seasonally because the rate and extent of starch gelatinisation, protein denaturation, and cell-wall disruption during the initial heating phase are influenced by ambient starting temperature and heating rate, thereby altering the substrate landscape available for both enzymatic and non-enzymatic transformation. These converging factors collectively shape the distinct metabolite fingerprints observed between winter and summer Daqu.

#### 3.2.4. Differential Pathway Analysis

**Figure 5:**
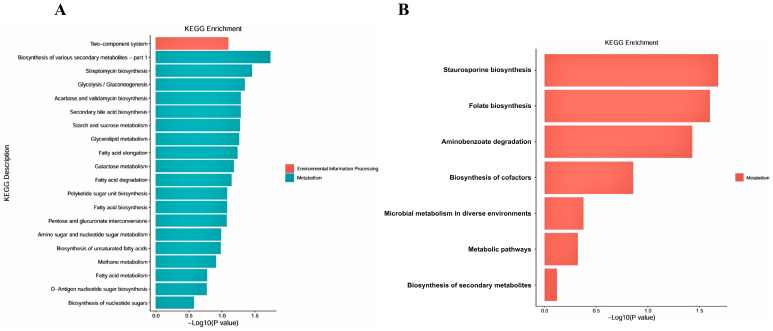
(**A**) KEGG enrichment of upregulated differential metabolites in winter Daqu. (**B**) KEGG enrichment of upregulated differential metabolites in summer Daqu.

Pathway enrichment based on differential metabolites (Figure 5) revealed contrasting metabolic strategies between seasons. In winter Daqu, the most significantly enriched upregulated pathways were biosynthesis of various secondary metabolites—part 1, streptomycin biosynthesis, and glycolysis. These pathways converge on secondary metabolism and basal carbon metabolism, suggesting that winter microbial metabolism is centred on energy provision and competitive regulation. The marked enrichment of glycolysis indicates enhanced carbon-source breakdown and patterns consistent with altered carbon distribution under low-temperature conditions; glycolysis not only supplies ATP and reducing equivalents but also generates precursors for fatty acid and amino acid biosynthesis, reflecting an ecological strategy of reinforcing basal metabolism to maintain energy homeostasis [36]. The enrichment of metabolites annotated to streptomycin biosynthesis and related secondary-metabolite pathways in winter Daqu may reflect increased pools of aminoglycoside-related intermediates; however, this pathway annotation should be interpreted cautiously, as the KEGG pathway mapping is based on metabolite structural similarity rather than confirmed biosynthetic activity. Whether these metabolites represent genuine antibiotic intermediates or structurally related compounds derived from alternative routes (e.g., sugar-nucleotide metabolism) remains to be established through targeted pathway validation [37].

In summer Daqu, the most significantly enriched upregulated pathways were staurosporine biosynthesis, folate biosynthesis, and aminobenzoate degradation. These pathways centre on cofactor biosynthesis and aromatic-compound metabolism, reflecting heightened metabolic activity and expanded compound-conversion capacity. The enrichment of folate biosynthesis-related metabolites in summer Daqu is consistent with, but does not directly demonstrate, elevated one-carbon metabolism and active cell proliferation. While folate is essential for nucleotide biosynthesis and thus correlates with growth activity in many microbial systems, alternative explanations—such as differential folate degradation rates at different temperatures or compositional shifts in folate-producing versus folate-consuming organisms—cannot be excluded without complementary growth-rate measurements [38]. Aminobenzoate degradation indicates enhanced capacity to metabolise aromatic compounds [39]; plant-derived aromatic substrates from the raw materials may be converted to carbon sources or flavour precursors, potentially expanding the precursor pool and metabolic network of summer Daqu.

The pathway enrichment patterns are consistent with a working hypothesis in which winter Daqu microbial communities prioritise basal energy metabolism and secondary metabolite accumulation, while summer communities exhibit pathway signatures associated with active growth and expanded biotransformation capacity. However, these designations remain interpretive frameworks rather than experimentally validated physiological states and should be tested through direct measurements such as biomass accumulation rates, ATP/ADP ratios, or growth-phase markers in future studies. These contrasting strategies are consistent with an adaptive reconfiguration of microbial energy utilisation and ecological regulation in response to seasonal temperature shifts.

### 3.3. Integrated DIA Quantitative Metaproteome–Metabolome Analysis of Functional Enzyme Expression Profiles and Non-Volatile Metabolites

DIA-based quantitative metaproteomics identified a total of 12,109 protein groups (corresponding to 45,329 unique peptides) across all samples, with an average of 10,985 proteins per sample. Taxonomic assignment of the identified proteins revealed that the dominant microbial contributors were *Aspergillus* spp. (8.7%) and *Bacillus* spp. (0.5%), followed by *Babjeviella* spp. (0.05%), *Ascosphaera* spp. (0.25%), and *Apophysomyces* spp. (0.18%). The remaining proteins were distributed among 98 additional genera (a total of 108 genera were identified at the genus level). Protein-level biological replicates (n = 3 per season) showed Pearson correlation coefficients > 0.85 within groups, confirming good reproducibility.

**Figure 6:**
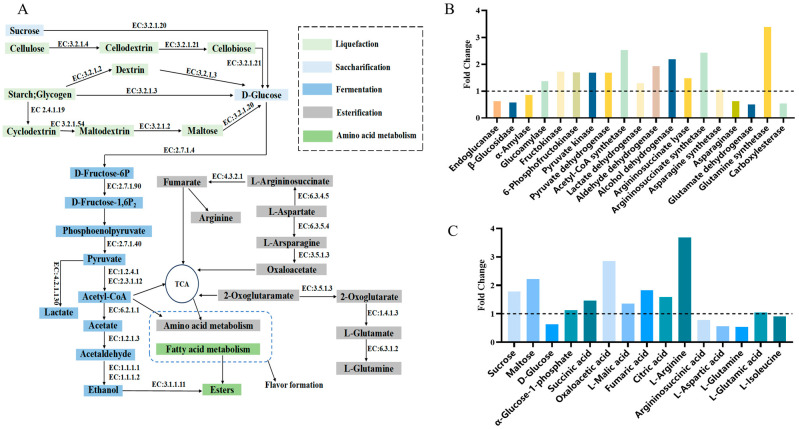
(**A**) Enzymes involved in selected metabolic pathways of medium-high-temperature Daqu. Colored boxes denote pathway types: light green = liquefaction, pale blue = saccharification, medium blue = fermentation, gray = esterification, dark green = amino acid metabolism. (**B**) Differential analysis of pathway-related metabolic enzymes between winter and summer. (**C**) Differential analysis of pathway-related metabolites between winter and summer.

To elucidate the functional drivers of seasonal differences in saccharification, fermentation and amino-acid metabolism in medium-high-temperature Daqu, DIA-based quantitative metaproteomics was used, in parallel with metabolomics, to systematically compare the relative abundances of key catalytic proteins between winter and summer samples. Combined with CAZy and KEGG annotation, this enabled construction of an integrated “functional enzyme protein expression—metabolic flux—non-volatile metabolite accumulation” framework (Figure 6A). It should be emphasised that all enzyme-level differences reported below are based on protein relative abundance (DIA intensity) rather than direct enzymatic activity assays or metabolic flux measurements. Protein abundance reflects the cumulative outcome of transcription, translation, and degradation, and does not necessarily correlate linearly with in situ catalytic activity, which is further modulated by post-translational modifications, substrate availability, cofactor concentrations, pH, and temperature. Consequently, the term ‘differential enzymes’ hereafter refers to differentially abundant enzyme proteins, which serve as proxies of catalytic potential. The enzyme–metabolite associations described below represent correlative patterns consistent with known biochemical pathways, rather than demonstrated causal relationships.

The enzymes presented in Figure 6 were identified through the following workflow: (i) all significantly differential proteins (FC > 1.5 or <0.67, Q < 0.05) were annotated against the CAZy, GO, and KEGG databases; (ii) proteins annotated to carbohydrate metabolism (glycolysis/gluconeogenesis, starch/sucrose metabolism), TCA cycle, and amino acid metabolism KEGG pathways were extracted; (iii) within these pathways, enzymes with established catalytic roles in substrate conversion relevant to flavour precursor formation were highlighted for integrated analysis with the corresponding metabolomic data. This selection was performed post-hoc based on functional annotation rather than a priori hypothesis.

Fold change (FC) was calculated to quantify the seasonal abundance difference of target proteins and metabolites, using summer Daqu as the reference control, with the formula: FC = mean abundance in winter Daqu/mean abundance in summer Daqu. Under this definition, FC > 1 indicates significantly higher abundance in winter Daqu, while FC < 1 indicates higher abundance in summer Daqu. As shown in Figure 6B,C, summer Daqu exhibited elevated abundances of endoglucanase, β-glucosidase, fructokinase, 6-phosphofructokinase, pyruvate dehydrogenase, acetyl-CoA synthetase, and arginine-metabolism-related enzymes, paralleled by elevated levels of maltose, D-fructose-6-phosphate, lactate, acetate, α-aminobutyrate, L-glutamate, L-aspartate, and flavour-related esters. These enzyme–metabolite pairs are jointly mapped onto glycolysis, the TCA cycle, amino acid metabolism, and downstream flavour-formation routes. Conversely, winter Daqu showed higher abundances of glucose oxidase, α-glucosidase, phosphoenolpyruvate carboxykinase, aldehyde dehydrogenase, and aspartate-related enzymes, accompanied by higher levels of maltodextrin, D-glucose-1,6-bisphosphate, fumarate, L-glutamate, oxaloacetate, citrate, and L-arginine, primarily reflecting carbohydrate storage, TCA-cycle intermediate accumulation, and amino-acid biosynthesis.

Compared with winter Daqu, summer Daqu thus exhibited stronger expression of polysaccharide-degrading and substrate-releasing enzymes, implying greater availability of fermentable sugars for downstream microbial utilisation. Based on the taxonomic annotation of our DIA metaproteomic data, the saccharolytic and glycolytic enzymes enriched in summer Daqu were predominantly assigned to thermotolerant bacterial genera—particularly *Bacillus* (contributing amylases, glucosidases, and proteases), *Lactobacillus* (responsible for lactate dehydrogenase and acetyl-CoA-related enzymes), and *Thermoactinomyces*—as well as fungal genera including *Aspergillus* and *Rhizopus* (contributing endoglucanases and β-glucosidases). These microorganisms utilise the released fermentable sugars through glycolysis to generate pyruvate, which is subsequently channeled into lactate fermentation (by *Lactobacillus* spp.), ethanol production (by *Saccharomyces* and *Pichia* spp.), or acetate and acetyl-CoA generation (by *Bacillus* and acetic acid bacteria), providing direct precursors for downstream ester and organic acid formation during Baijiu fermentation.

At the level of central carbon metabolism, the enhanced abundances of pyruvate dehydrogenase, acetyl-CoA synthetase and lactate dehydrogenase corresponded to elevated lactate, acetate and acetyl-CoA-derived intermediates, consistent with active glycolysis–TCA-cycle flux that provides energy and direct precursors for fatty acid, alcohol and organic acid biosynthesis. At the amino acid level, summer Daqu was enriched in L-arginine, L-glutamate, fumarate and related intermediates, supporting downstream aroma and flavour-complexity formation through amino-acid–organic-acid interactions and Maillard chemistry. By contrast, winter Daqu, with comparatively lower expression of saccharolytic and central metabolic enzymes, accumulated upstream intermediates and storage-type carbohydrates, indicating relatively constrained substrate supply and flavour-precursor formation under low-temperature conditions.

Taken together, the proteome–metabolome integration analysis demonstrates that the seasonal divergence of non-volatile metabolic profiles is correlatively associated with a coherent reconfiguration of in situ functional enzyme expression. Summer production conditions favour higher-expression-level deployment of saccharolytic, glycolytic and amino-acid-converting enzymes, coinciding with greater accumulation of fermentable substrates, central-metabolic intermediates and amino-acid precursors—hallmarks of a flavour-precursor-rich state. Winter production conditions, in contrast, favour a conservative carbon-storage and energy-maintenance enzyme repertoire. Overall, the integrated analysis indicates that seasonal factors reshape Daqu quality primarily by modulating the in situ expression landscape of key metabolic enzyme proteins, with summer Daqu showing a clear advantage in saccharolytic enzyme expression and flavour-precursor accumulation.

A limitation of the present study is the absence of dedicated amplicon-based community profiling (16S rRNA/ITS sequencing). However, the taxonomic annotations derived from DIA metaproteomics offer a complementary perspective by identifying metabolically active organisms rather than total community membership. Future studies integrating amplicon sequencing with the metaproteomic and metabolomic data presented here would enable a more comprehensive ‘community structure–functional expression–metabolite output’ framework.

Based on these pathway enrichment patterns, we propose—as a working hypothesis to guide future investigation—that winter Daqu microbial metabolism may be characterised by a ‘basal-maintenance-like’ pattern, while summer Daqu shows signatures more consistent with an ‘active-metabolism-like’ pattern. These descriptors are offered as conceptual shorthand for the observed metabolite distribution patterns and should not be equated with experimentally determined physiological states such as growth rate or metabolic flux measurements.

## 4. Conclusions

This study applied the dual-omics characterisation of seasonal divergence in medium-high-temperature Daqu by integrating DIA-based quantitative metaproteomics with untargeted LC-MS metabolomics. At the metabolome level, 2904 non-volatile metabolites were annotated and 1472 differential metabolites (560 upregulated and 912 downregulated in winter versus summer) were resolved, predominantly comprising lipids and lipid-like molecules, organoheterocyclic compounds, and organic acids and derivatives; PCA and OPLS-DA models confirmed robust seasonal separation. Pathway enrichment further indicated that winter Daqu adopted a metabolic-maintenance configuration, with upregulated glycolysis and secondary metabolite biosynthesis under low-temperature conditions, whereas summer Daqu exhibited a rapid-growth phenotype characterised by enhanced folate biosynthesis, aromatic-compound degradation, and active central carbon metabolism. At the metaproteome level, summer Daqu was enriched in saccharolytic enzymes (endoglucanase, β-glucosidase), glycolytic enzymes (fructokinase, 6-phosphofructokinase, pyruvate dehydrogenase), and acetyl-CoA-, lactate-, and amino-acid-related enzymes, consistent with elevated maltose, D-fructose-6-phosphate, lactate, acetate, and key amino acids (L-glutamate, L-aspartate, α-aminobutyrate); conversely, winter Daqu showed higher abundance of glucose oxidase, α-glucosidase, phosphoenolpyruvate carboxykinase, aldehyde dehydrogenase, and aspartate-related enzymes, mirroring the accumulation of maltodextrin, glucose-1,6-bisphosphate, fumarate, oxaloacetate, citrate, and L-arginine and reflecting a conservative carbon-storage configuration. Therefore, the proteome–metabolome correspondence supports an “enzyme expression–substrate conversion–non-volatile metabolite accumulation” axis as the proximate molecular basis of seasonal Daqu quality divergence, with summer production conditions favouring a more flavour-precursor-rich state. The DIA metaproteome–metabolome integrated analysis framework established in this study provides a correlative reference for understanding the seasonal compositional divergence of medium-high-temperature Daqu and identifies a list of candidate functional proteins (including saccharolytic glycoside hydrolases, central carbon metabolism-related kinases, and amino acid conversion enzymes) whose differential seasonal expression corresponds to distinct non-volatile metabolite profiles. These candidate enzymes represent potential markers and testable hypotheses for future research aimed at screening high-enzyme-expression starter strains and developing season-adaptive production protocols. Validation of these candidates through direct enzyme activity measurement, pilot-scale fermentation, and sensory evaluation of the resulting Baijiu will be essential to establish whether and how the observed Daqu-level differences propagate to final product quality.

Furthermore, a methodological limitation of the metabolomics analysis should be acknowledged. Owing to the small number of samples (only three biological replicates per season), no independent pooled QC sample was analysed for routine quality control procedures (e.g., RSD-based filtering). Data quality was instead assessed by the tight clustering of biological replicates in PCA and high intra-group Pearson correlations (>0.85), and the key metabolic differences were independently supported by the metaproteomic data. Nevertheless, the absence of a standard QC workflow may affect the precise evaluation of quantitative reproducibility for low-abundance metabolites. Future studies will adhere to the Metabolomics Standards Initiative (MSI) guidelines by incorporating pooled QC samples and systematic RSD filtering to improve the rigor and reliability of metabolomics reporting.

Several limitations should be noted. First, protein relative abundance only reflects catalytic potential rather than actual in-situ enzyme activity. To validate the functional significance of the differentially expressed proteins identified here, future work should incorporate targeted enzyme activity assays for industry-relevant metrics, including saccharification power and esterification capacity. Second, this study only characterised the Daqu starter itself and did not track how seasonal differences in enzyme and metabolite profiles propagate through downstream Baijiu fermentation to shape the volatile aroma profile and sensory quality of the final product. Essential follow-up work will include pilot-scale fermentation trials using winter and summer Daqu, coupled with volatile profiling via GC-MS/GC-O and quantitative sensory evaluation of the resulting Baijiu, to clarify the transmission mechanism of quality differences from Daqu to the final product. Third, this study did not report conventional physicochemical quality parameters of Daqu (saccharification power, fermentation power, esterification power, acidity, moisture content). Future studies should integrate these industry-standard metrics to bridge omics-level molecular observations with routine quality control indicators used in Baijiu production.

## Data Availability

The original contributions presented in this study are included in the article. Further inquiries can be directed to the corresponding authors.

## References

[1] Wu Q., Zhu Y., Fang C., Wijffels R.H., Xu Y. (2021). Can We Control Microbiota in Spontaneous Food Fermentation?–Chinese Liquor as a Case Example. Trends Food Sci. Technol..

[2] Tang Q., Zhang Y., Huang J., Zhou R. (2025). Unraveling the Unique Microbiota and Metabolites in Three Different Colors Jiangqu through Multidimensional Analysis. Food Chem..

[3] Deng J., Zheng J., Huang D., Huang Z., Ye G., Luo H. (2023). Characterization of physicochemical properties, volatile compounds and microbial community structure in four types of Daqu. LWT.

[4] Shi G., Fang C., Xing S., Guo Y., Li X., Han X., Lin L., Zhang C. (2024). Heterogenetic Mechanism in High-Temperature Daqu Fermentation by Traditional Craft and Mechanical Craft: From Microbial Assembly Patterns to Metabolism Phenotypes. Food Res. Int..

[5] Tang Q., Zhang Y., Huang J., Zhou R. (2025). Synthetic and Natural Microbial Communities in High-Temperature Daqu Production: Insights into Metabolic Pathways and Volatile Organic Compounds. Food Res. Int..

[6] Yang L., Fan W., Xu Y. (2024). Effects of Storage Period and Season on the Microecological Characteristics of Jiangxiangxing High-Temperature Daqu. Food Res. Int..

[7] Zhang Y., Zhang Z., Huang J., Zhou R., Tang Q., Jin Y. (2024). Characterizing the Contribution of Strain Specificity to the Microbiota Structure and Metabolites of Muqu and Fresh High-Temperature Daqu. Foods.

[8] Xiao C., Lu Z., Zhang X., Wang S., Ao L., Shen C., Shi J., Xu Z. (2017). Bio-Heat Is a Key Environmental Driver Shaping the Microbial Community of Medium-Temperature Daqu. Appl. Environ. Microbiol..

[9] Wang L., Cheng Y., Hu X., Huang Y. (2022). Analysis of Bacterial Diversity and Functional Differences of Jiang-Flavored Daqu Produced in Different Seasons. Front. Nutr..

[10] Wu S., Du H., Xu Y. (2023). Daqu Microbiota Adaptability to Altered Temperature Determines the Formation of Characteristic Compounds. Int. J. Food Microbiol..

[11] Lei Z., Zhang Z., Huang J., Tang Q., Zhou R. (2025). Revealing the Synergistic Effects of Qupi-Shaping Pattern and Spatial Variation on High-Temperature Daqu Attributes. LWT.

[12] Jiang X., Peng Z., Zhu Q., Zheng T., Liu X., Yang J., Zhang J., Li J. (2023). Exploration of Seasonal Fermentation Differences and the Possibility of Flavor Substances as Regulatory Factors in Daqu. Food Res. Int..

[13] Pan Z., Cao M., Jiang J., Yue K. (2025). Unveiling Microgeographical and Seasonal Variation in the Microbiome and Proteome of Fen-Flavored Daqu. Food Biosci..

[14] Zhao J., Yang Y., Chen L., Zheng J., Lv X., Li D., Fang Z., Shen C., Mallawaarachchi V., Lin Y. (2022). Quantitative Metaproteomics Reveals Composition and Metabolism Characteristics of Microbial Communities in Chinese Liquor Fermentation Starters. Front. Microbiol..

[15] Wu X., Zhao X., Wang L., Chen B., Li F., Tang Z., Wu F. (2024). Unraveling the Regional Environmental Ecology Dominated Baijiu Fermentation Microbial Community Succession and Associated Unique Flavor. Front. Microbiol..

[16] Tang Q., Chen X., Huang J., Zhang S., Qin H., Dong Y., Wang C., Wang X., Wu C., Jin Y. (2023). Mechanism of Enhancing Pyrazines in Daqu via Inoculating *Bacillus licheniformis* with Strains Specificity. Foods.

[17] Niu Y., Zhao W., Xiao Z., Zhu J., Xiong W., Chen F. (2023). Characterization of Aroma Compounds and Effects of Amino Acids on the Release of Esters in Laimao Baijiu. J. Sci. Food Agric..

[18] Zhao Y., Han Y., Cao R., Xu Y., Mu X. (2025). Multiomics Insights into Microbiome Succession throughout Medium-Temperature Daqu Fermentation. Food Sci. Technol..

[19] Zhou Z., Liu S., Zhu Y., Liu S., Ji Z., Ren Q., Pan X., Mao J. (2024). Effect of Caramel Colors Addition on the Color, Physicochemical Properties, and Flavor of Huangjiu. J. Food Compos. Anal..

[20] Gouda H., Agongo J., Caraballo-Rodríguez A.M., Dorrestein P.C. (2025). The Mass Spectrometry of Microbiome-Mediated Metabolism of Food: Challenges and Opportunities. Curr. Opin. Microbiol..

[21] Yang L., Fan W., Xu Y. (2021). GC × GC-TOF/MS and UPLC-Q-TOF/MS Based Untargeted Metabolomics Coupled with Physicochemical Properties to Reveal the Characteristics of Different Type Daqus for Making Soy Sauce Aroma and Flavor Type Baijiu. LWT.

[22] Zhu M., Deng Z., Tie Y., Quan S., Zhang W., Wu Z., Pan Z., Qin J., Wu R., Luo G. (2024). Unveiling the Synthesis of Aromatic Compounds in Sauce-Flavor Daqu from the Functional Microorganisms to Enzymes. Food Res. Int..

[23] Tan Y., Zhu Y., Wijffels R.H., Zhang H., Scott W.T., Xu Y., dos Santos V.M. (2025). Controlling Metabolic Stability of Food Microbiome for Stable Indigenous Liquor Fermentation. npj Biofilms Microbiomes.

[24] Hu J., Sun J., Shu X., Du H., Xu Y. (2026). Revealing the Key Core Microbiota of High-Temperature Daqu and the Contact-Dependent Characteristics of Growth Promotion. Int. J. Food Microbiol..

[25] Hu X., Tian R., Fan J., Han S., Li J., Li H., Wang Y., He P. (2022). Research Progress on the Aroma Contribution and Their Regional Characteristics of Volatile Compounds in Chinese Strong-Flavor Baijiu. J. Light Ind..

[26] Ren H., Fan J., Guo X., Zhang B., Zhao H., Ma D., Liu S., Mao J., Zhang B., Qiao J. (2026). Deciphering the Characteristics of Strong-Flavor Daqu at Different Grades through Integrated Microbiome and Metabolome Analysis. Food Chem. X.

[27] Chen J., Wang W., Li H., Wang F., Fu L., Dong K., Jia L., Wang Y. (2022). Progress in Multi-Omics Research on Flavor Formation Mechanism in Fermented Aquatic Foods. Meat Res..

[28] Du Y., Tang J., Liu D., Liu N., Peng K., Wang C., Huang D., Luo H. (2023). Microbial Metabolism during the Thermophilic Phase Promotes the Generation of Aroma Substances in Nongxiangxing Daqu. Food Chem. X.

[29] Yuan B., Jin H., Pian L., Gao S., Huang Z. (2024). A Review of the Impact of Maillard Reaction on Food Quality and Safety and the Detection of Its Products. J. Light Ind..

[30] Yang L., Fan W., Xu Y. (2023). Chameleon-Like Microbes Promote Microecological Differentiation of Daqu. Food Microbiol..

[31] Dong W., Peng Y., Ma J., Hu Y., Chen S., Zhao S. (2025). Yeasts in Traditional Baijiu Fermentation: Diversity, Functions, Microbial Interactions and Applications. Front. Microbiol..

[32] Chen P., Feng X., Zhu Y., Wu J., Li H., Jiang S., Zhang Y., Liu Y., Zheng J., Sun J. (2023). Sweetness Science of Baijiu: Unraveling Flavor Compounds, Perception and Analytical Techniques. Food Chem. Adv..

[33] Huang H., Gao Y., Wang L., Yu X., Chen S., Xu Y. (2024). Maillard Reaction Intermediates in Chinese Baijiu and Their Effects on Maillard Reaction Related Flavor Compounds during Aging. Food Chem. X.

[34] Huang Z., Zeng B., Deng J., Ren Z., Xie J., Wei C. (2024). Succession of Microbial Community Structure in Fermented Grains during the Fermentation of Strong-Flavor Baijiu and Its Impact on the Metabolism of Acids, Alcohols, and Esters. Food Sci. Biotechnol..

[35] Li M., Shen Y., Ling T., Ho C.-T., Li D., Guo H., Xie Z. (2021). Analysis of Differentiated Chemical Components between Zijuan Purple Tea and Yunkang Green Tea by UHPLC-Orbitrap-MS/MS Combined with Chemometrics. Foods.

[36] Zong E., Bo T., Dang L., Zhang J., Li H., Lv N., He Y., Bai B., Zhang J., Fan S. (2024). Different Functions Can Be Provided by Low Temperature Daqu with Different Appearance Features Due to Variations in the Microbial Community Structure during Fermentation. LWT.

[37] Pócsi I., Dijksterhuis J., Houbraken J., de Vries R.P. (2024). Biotechnological Potential of Salt Tolerant and Xerophilic Species of *Aspergillus*. Appl. Microbiol. Biotechnol..

[38] Kok D.E., Steegenga W.T., Smid E.J., Zoetendal E.G., Ulrich C.M., Kampman E. (2020). Bacterial Folate Biosynthesis and Colorectal Cancer Risk: More Than Just a Gut Feeling. Crit. Rev. Food Sci. Nutr..

[39] Hou M., Martin J.J.J., Li W., Dong Z., Wang Q., Chen Y., Song Y., Sun C., Cao H. (2025). Comprehensive Metabolite Profiling Reveals Biochemical Evolution in Coconut Water during Fruit Development. Sci. Hortic..

